# Missing Numbers

**Published:** 1885-10

**Authors:** 


### MISSING NUMBERS.

Owing to an unusual demand, the edition of the number of this Journal for February, 1885, is entirely exhausted. We will send valuable reprints, or any other number, or will pay cash for copies of that number. Any one having such, which he does not care to preserve, will confer a great favor by mailing them to the editor at Buffalo.

---

### MORE DENTAL COLLEGES.

Two new colleges have been chartered for Chicago. They will be welcomed if the only emulation is to see which shall turn out the best, instead of the greatest number of students.

